# Acute pancreatitis as a clinical presentation of COVID-19 in a patient with HIV infection: a case report

**DOI:** 10.1093/gastro/goac003

**Published:** 2022-02-09

**Authors:** Justyna D Kowalska, Carlo Bieńkowski, Beata Wojtycha-Kwaśnica, Pawel Uliczny, Andrzej Horban

**Affiliations:** 1 Department of Adults’ Infectious Diseases, Medical University of Warsaw, Hospital for Infectious Diseases, Warsaw, Poland; 2 Department of Diagnostic Imaging, Hospital for Infectious Diseases in Warsaw, Warsaw, Poland; 3 Emergency Department, Hospital for Infectious Diseases in Warsaw, Warsaw, Poland

## Background

Severe acute respiratory syndrome coronavirus 2 (SARS-CoV-2) occurs mostly as a respiratory tract infection; however, a substantial proportion of patients present with other symptoms [1]. In order to enter a cell, the SARS-CoV-2 virus requires an angiotensin-converting enzyme 2 (ACE2) transmembrane protein (ACE2 receptor). ACE2 receptors are >100 times more common in the gastrointestinal (GI) tract than in the respiratory tract, and many SARS-CoV-2-infected patients present with GI symptoms [2]. In the course of coronavirus disease 2019 (COVID-19), cytopathogenic T-cells and activated monocytes may enter the GI tract in the same way as they do in pulmonary circulation and initiate the inflammation leading to organ injury and immune disorders. Pancreatic cells present a high expression of ACE2 receptors and this creates the potential for pancreatic injury during COVID-19 [3]. However, to date, only a few COVID-19-related cases of pancreatitis have been reported, and none in HIV-positive persons [4, 5]. Currently, the incidence of pancreatitis and its future clinical implications for HIV-positive patients remain unknown.

## Case presentation

A 26-year-old man infected with HIV in 2016 through having sex with men (MSM) was consulted in the Emergency Department (ER) of the Hospital for Infectious Diseases in Warsaw (Poland) due to a 5-day history of loss of smell, a dry cough and myalgia, and a 2-day history of dyspnea. He was on stable antiretroviral treatment with dolutegravir, tenofovir alafenamide, and emtricitabine. His most recent lymphocyte CD4^+^ cell count was 1,610 cells/mm^3^, his CD4^+^ percentage was 55%, and his HIV viral load (VL) was undetectable. Both hepatitis B and C infections were excluded. In relation to lifestyle, he had a well-balanced diet, he was a non-smoker, reported occasional use of alcohol, and denied any use of illicit psychoactive substances. However, a week before the onset of symptoms, he met with a few friends and had a cannabis cookie.

On physical examination, no significant abnormalities were present. A nasal swab was performed and the reverse transcriptase–polymerase-chain-reaction (RT-PCR) assay confirmed a SARS-CoV-2 infection. He was diagnosed with mild COVID-19 and referred for home isolation.

Two weeks later (3 weeks after his initial symptoms), he returned to the ER with a 3-day history of pain in the upper abdomen and nausea. He had no symptoms of respiratory tract infection and his peripheral blood oxygenation was SaO_2_ 96%. He was admitted to the hospital and diagnosed with acute pancreatitis based on a serum lipase value of 5,855 U/L (reference range, 23–300 U/L) and a serum amylase value of 350 U/L (reference range, 30–110 U/L) (further laboratory test results are shown in Table 1). The results of his chest X-ray were normal. The abdominal ultrasound showed a normal-sized, slightly non-homogeneous pancreas without any focal lesion or fluid collection. The common biliary duct was not dilated and there were no gallstones found. There were no changes found in other organs and no ascitic fluid.

**Table 1.** Patient’s laboratory test results on admission, and after 2 and 6 weeks of being discharged home

Laboratory test	Reference range	On admission	2 weeks later	6 weeks later
Lipase, U/L	23–300	5,855	1,729	33
Amylase, U/L	30–110	350	78	30
Total bilirubin, µmol/L	3–22	5	5	7.9
Alanine transaminase, U/L	4–35	17	21	23
Aspartate transaminase, U/L	10–36	24	24	19
Gamma-glutamyltransferase, U/L	12–43	19	19	20
C-reactive protein, mg/L	<10	53	32	<5
D-dimers, ng/mL	<500	892	654	<500
White blood cell count, /mm^3^	4,000–10,000	10,100	9,800	9,200
Neutrophils percentage, %		53.2	58.9	48.6
Lymphocyte CD4^+^ count, cells/µL		1,610 (the most recent before admission)	987	1,102
Lymphocyte CD4^+^ percentage, %		55	55	55
Calcium level, mmol/L	2.10–2.55	2.35	NA	NA
Total cholesterol, mmol/L	<5	4.71	NA	NA
Low-density lipoprotein level, mmol/L	<2.5	2.76	NA	NA
High-density lipoprotein level, mmol/L	≥1	1.19	NA	NA
Triglycerides, mmol/L	<1.7	1.14	NA	NA
Symptoms		Nausea Abdominal pain No respiratory tract symptoms	Nausea Abdominal pain No respiratory tract symptoms	No symptoms

NA, not available.

A contrast-enhanced computed tomography (CECT) was also carried out and showed that the pancreas was not enlarged, had no focal lesion, and had a subtle obliteration of the lobar structure, with a gentle homogeneous enhancement. The common biliary duct was not dilated. Infiltration of the peripancreatic fat was present, and several mesenteric and peripancreatic lymph nodes were enlarged by ≤6 mm (short axis). No other abnormalities were found.

During hospitalization, the patient was tested twice for SARS-CoV-2 using a nasopharyngeal swab with a RT-PCR test: both results were negative. After 4 days of conservative treatment, the patient was referred home where he remained under the supervision of the outpatient clinic.

Two weeks later, on the first follow-up visit, the patient reported persistent pain in the upper abdomen and problems with tolerating normal food (Table 1).

On the next visit, 6 weeks after being discharged, follow-up testing revealed undetectable HIV VL, a decrease in the lymphocytes CD4^+^ count to 1,102 cells/mm^3^ (55%), and a 1.3 CD4^+^/CD8^+^ ratio (Table 1).

## Discussion and conclusions

In retrospective analyses by Pan *et al.* [6], more than half of the patients hospitalized in Union Hospital, Tongji Medical College (Wuhan, China) with mild COVID-19 had both respiratory and GI symptoms, while >20% had GI symptoms only. Patients with GI symptoms were more likely to report for medical care later. Our case presents a patient with both respiratory and GI symptoms; the latter, however, occurred 3 weeks after the first symptoms. Nevertheless, some case reports indicate that GI symptoms may be present before respiratory symptoms [7].

Antiretroviral drug-induced pancreatitis may be observed in HIV-positive patients, although it is highly unlikely in the present case as the patient had been on well-tolerated cART since 2016 [8]. Whether, and to what extent, HIV remains a factor in the course and clinical presentation of SARS-CoV-2 infection remains unknown [9].

Peluso *et al.* [10] discussed the HIV VL increase in the course of COVID-19 in HIV-positive patients. Their analysis included 12 patients and revealed that VL had increased in 10 patients with SARS-CoV-2/HIV coinfection; however, there was no statistical significance between COVID-19-positive and COVID-19-negative participants. In our case report, the patient’s HIV VL remained undetectable during both the course of the disease and the follow-up visits.

Akkus *et al.* [11] retrospectively investigated a group of 127 patients who had increased lipase levels during the course of COVID-19. The study revealed that the risk of developing elevated pancreatic enzymes was high in patients with SARS-CoV-2 infection, especially in those with pre-existing diabetes.

Some recent reports suggest that HIV-positive patients may experience HIV VL blips; however, we did not observe this in our patient. Although his CD4^+^ cell count dropped, it was within the normal range, with a high CD4^+^/CD8^+^ ratio and stable CD4^+^ percentage [10].

To conclude, the symptomatology of COVID-19 is very broad and may be related to injury to a range of organs. From the pathophysiological perspective, the involvement of the pancreas is plausible but is rarely diagnosed in clinical practice. Whether the rare involvement of the pancreas is related to the focus on respiratory symptoms and under-diagnosis or is clinically factual remains unclear. Therefore, in COVID-19 cases, serum lipase levels should be considered a standard laboratory test and be included in the routine laboratory tests panel. Abdominal ultrasound and CECT should be considered diagnostic tools in patients with abnormal laboratory findings or clinical manifestations suggesting pancreatic involvement.

## Authors' Contributions

J.D.K. and C.B. drafted the manuscript, and analysed and interpreted the data; J.D.K., B.W.K., and P.U. collected the data; and J.D.K. and A.H. revised the manuscript. All authors read and approved the final manuscript.

## Funding

Foundation for Science Development in the Hospital for Infectious Diseases in Warsaw, Poland (Fundacja Rozwoju Nauki w Wojewódzkim Szpitalu Zakaźnym w Warszawie) funded the article processing charges.
